# Resveratrol Mediates the Apoptosis of Triple Negative Breast Cancer Cells by Reducing POLD1 Expression

**DOI:** 10.3389/fonc.2021.569295

**Published:** 2021-02-25

**Authors:** Zhi-Jie Liang, Yan Wan, Dan-Dan Zhu, Meng-Xin Wang, Hong-Mian Jiang, Dong-Lin Huang, Li-Feng Luo, Mao-Jian Chen, Wei-Ping Yang, Hong-Mian Li, Chang-Yuan Wei

**Affiliations:** ^1^ Department of Breast Surgery, Guangxi Medical University Cancer Hospital, Nanning, China; ^2^ Department of Wound Repair Surgery, The Fifth Affiliated Hospital of Guangxi Medical University & The First People’s Hospital of Nanning, Nanning, China; ^3^ Department of Pathology, The Fifth Affiliated Hospital of Guangxi Medical University & The First People’s Hospital of Nanning, Nanning, China; ^4^ Department of Plastic and Aesthetic Surgery, The Fifth Affiliated Hospital of Guangxi Medical University & The First People’s Hospital of Nanning, Nanning, China; ^5^ Department of Ultrasonography, Guangxi Medical University Cancer Hospital, Nanning, China

**Keywords:** resveratrol, triple negative breast cancer, apoptosis, RNA-seq, POLD1

## Abstract

Resveratrol (RSV) is known to possess anticancer properties in many types of cancers like breast cancer, in which POLD1 may serve as a potential target. However, the anticancer mechanism of RSV on triple negative breast cancer (TNBC) remains unclear. In the present study, the antitumor effects and mechanism of RSV on TNBC cells were analyzed by RNA sequencing (RNA-seq), which was then verified *via* cell counting kit-8 (CCK8), immunofluorescence, immunohistochemistry, Western Blot (WB), flow cytometry, and hematoxylin-eosin (HE) staining. According to the corresponding findings, the survival rate of MDA-MB-231 cells gradually decreased as RSV treatment concentration increased. The RNA-seq analysis results demonstrated that genes affected by RSV treatment were mainly involved in apoptosis and the p53 signaling pathway. Moreover, apoptosis of MDA-MB-231 cells induced by RSV was observed to be mainly mediated by POLD1. When treated with RSV, the expression levels of full length PARP1, PCNA, and BCL-2 were found to be significantly reduced, and the expression level of Cleaved-PARP1 as well as Cleaved-Caspase3 increased significantly. Additionally, the mRNA expression of POLD1 was significantly reduced after treatment with RSV, and the protein expression level was also inhibited by RSV in a concentration-dependent manner. The prediction of domain interaction suggested that RSV may bind to at least five functional domains of the POLD1 protein (6s1m, 6s1n, 6s1o, 6tny and 6tnz). Furthermore, after RSV treatment, the anti-apoptotic index (PCNA, BCL-2) of MDA-MB-231 cells was found to decrease while the apoptosis index (caspase3) increased. Moreover, the overexpression of POLD1 reduced the extent of apoptosis observed in MDA-MB-231 cells following RSV treatment. Moreover, animal experimental results showed that RSV had a significant inhibitory effect on the growth of live tumors, while POLD1 overexpression was shown to antagonize this inhibitory effect. Accordingly, this study’s findings reveal that RSV may promote the apoptosis of TNBC cells by reducing the expression of POLD1 to activate the apoptotic pathway, which may serve as a potential therapy for the treatment of TNBC.

## Introduction

In 2018, there were about 2.1 million newly diagnosed female breast cancer cases globally, accounting for nearly a quarter of all female cancer cases (1). According to the expression levels of estrogen receptor [ER], progesterone receptor [PR], and human epidermal growth factor receptor 2 [HER2] protein, breast cancer can be divided into four subtypes: luminal -A, luminal-B, HER-2 positive, and triple negative breast cancer (TNBC) (2, 3). Among them, TNBC accounts for about 15% - 20% of breast cancer (4), and compared to hormonal receptor positive or HER2 positive diseases, TNBC is characterized by its highly aggressive clinical progression due to its earlier onset age, greater metastatic potential and worse clinical results (5, 6). TNBC progresses very rapidly, and due to the lack of common therapeutic targets, current methods in controlling disease development are very limited (7). TNBC patients usually receive anthracycline and cyclophosphamide chemotherapy, and subsequently take taxane (anthracycline/taxane [ACT]) as standard care. However, only about one-third of patients attain a pathological complete response (PCR), while remaining patients relapse and eventually succumb to the disease (8). Several studies have shown that resveratrol (RSV) possesses anticancer properties (9) in breast cancer (10) and may serve as a potential therapeutic candidate for TNBC.

RSV is a non-flavonoid polyphenolic compound produced by plants when treated. It is a bioactive component in wine and grape juice and can be synthesized in grape leaves and grape skins (11, 12). Research has shown that RSV has functions in anti-oxidation, anti-inflammatory, anti-cancer, and cardiovascular protection (11, 13–15). Moreover, RSV has a significant inhibitory effect on a variety of tumor cells, such as liver cancer, breast cancer, colon cancer, gastric cancer, and leukemia (9, 16). RSV can enhance cancer radiotherapy and effectively inhibit the role of cancer stem cells (17). For TNBC cells such as MDA-MB-231, RSV can inhibit its metastasis by reversing TGF-β1-induced epithelial matrix transformation (18). However, due to the complex anti-tumor mechanism of RSV, researchers have yet to reach a consensus on its mechanism of action, hence, the anti-cancer mechanism of RSV on TNBC remains unclear. In addition, the DNA polymerase delta catalytic subunit gene (POLD1) is one of the subunits of polδ in DNA polymerases (19). High expression or point mutations may lead to DNA replication and cell cycle abnormality of common cells, which may then progress to cancer cells. POLD has been confirmed to be regulated by a variety of upstream factors, such as p53 and SIRT1 (20, 21), which are the direct regulatory targets of RSV. However, no studies have discussed the regulation of POLD1 by RSV. Additionally, the role of POLD1 within RSV-mediated apoptosis of cancer cells is also unclear.

**Figure 1:**
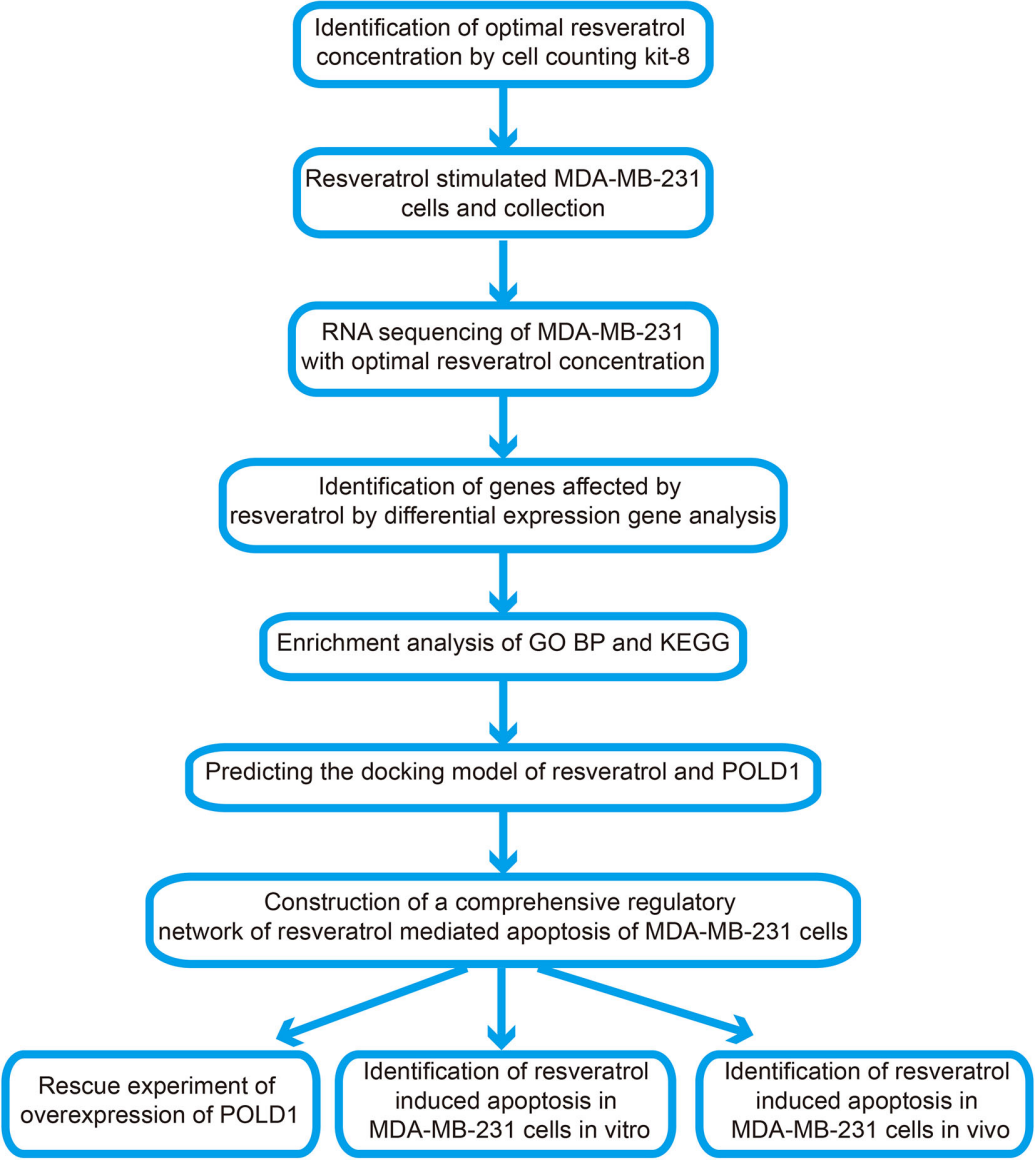
Flow chart of present study.

Therefore, this study aims to explore the specific mechanism surrounding RSV-mediated apoptosis of TNBC cells. The corresponding flow chart of this research is shown in **Figure 1**. RNA sequencing was used to observe the changes of POLD1, other apoptosis-related pathways and factors in MDA-MB-231 under RSV treatment. Additionally, genes and pathways related to POLD1’s potential role were analyzed, and changes in the transcription and translation levels of POLD1 and its related genes were verified. Subsequently, *via* ectopic expression of POLD1, the role of POLD1 in RSV-mediated breast cancer cell apoptosis *in vivo* and *in vitro* were further explored. The obtained results suggested that RSV may promote apoptosis of TNBC cells by reducing the expression of POLD1 to activate the apoptotic pathway, which may be a potential mechanism pertaining to its anticancer effects on TNBC.

## Materials and Methods

### Materials and Ethical Approval

In the Cancer Genome Atlas (TCGA, https://www.cancer.gov/) database, the transcriptome data of breast cancer (BRCA) samples and control samples were downloaded. Moreover, the TNBC cell lines MDA-MB-231 and common breast epithelial cell lines MCF10A were derived from the Shanghai Cell Bank of the Shanghai Academy of Sciences. BALB/c nude female mice (weight 18-22g, 4–6 weeks old) were obtained from the Animal Experiment Center of Guangxi Medical University. All experiments were approved by the Animal Ethics Approval Committee of Guangxi Medical University.

### Effect of RSV on MCF-10A and MDA-MB-231 Cell Survival

Cell Counting Kit-8 (CCK8) (Dojindo, Japan) was used to detect the effect of RSV (Solarbio, China) on the survival of MCF-10A and MDA-MB-231 cells. The cells in the logarithmic growth phase were adjusted to a concentration of 5 × 10^4^/ml and were inoculated in 96-well plates (100 µl per well). The experiment was divided into eight groups, and each group was set up with five duplicate wells that were incubated in a 37 °C, 5% CO_2_ incubator overnight. After the cells were adhered, the medium was removed, and 100 µl of DMEM (Gibco, USA) containing RSV at concentrations of 0, 3.125, 6.25, 12.5, 25, 50, 100, and 200 μM were added in groups. The control group had 100 µl of 0.1% DMSO in DMEM added, which was then incubated for 24 h under the same conditions. Next, 10 µl of CCK8 solution was added to each well and incubation continued for 3 h. A microplate reader was used to measure absorbance (A) at 450 nm. The survival rates of the eight groups of MCF-10A and MDA-MB-231 cells were then compared, and the experiment was repeated six times. The concentration of RSV that produced significant differences in survival rates between the two cell lines was chosen as the optimum concentration for further experiments.

### The Treatment of MDA-MB-231 Cells With Resveratrol

Here, 5 × 10^4^ cells/well of MDA-MB-231 cells in the logarithmic growth phase were taken and inoculated into 6-well plates, after which 10% FBS serum DMEM medium was added and incubated in a 37°C, 5% CO_2_ incubator. When MDA-MB-231 cells were observed to reach 80% confluence, PBS was used to wash the cells three times, and the serum-containing DMEM was replaced with serum-free DMEM. The cells were starved overnight and were then divided into the control group and RSV treatment group. On the next day, after removing the DMEM medium, a new serum-free DMEM medium was added to the control group, and an optimum concentration RSV containing DMEM medium was added to the RSV treatment group, which was then cultured in a 37°C, 5% CO_2_ incubator for 24 h. Subsequently, the culture medium was removed, and 1 ml of PBS was added to each well, followed by gentle washing and removal. After this operation was repeated twice, 1 ml of trypsin was added to each well and digested in a 5% CO_2_ incubator at 37°C for 1 to 1.5 min, and 2 ml of 10% FBS complete medium was added. Then, digestion was stopped, and all cells and liquid were collected into 15 ml centrifuge tubes.

### RNA Extraction and Sequencing

After treatment of MDA-MB-231 cells with resveratrol, RNA from MDA-MB-231 cells treated by the optimal concentration of resveratrol were extracted using Trizol. In order to ensure that the samples were qualified and appropriate for sequencing, Nanodrop, Qubit 2.0 and Aglient 2100 were used to detect the purity, concentration, and integrity of RNA samples, respectively. The optical density (OD) 260/280 of the sample must be greater than 1.8, and it must be free of protein, or visible impurities. Library construction and RNA sequences were performed according to the manufacturer’s instructions. RNA sequencing was performed on MDA-MB-231 cells using NovaSeq. After RNA-seq, the clean data was first filtered to remove low-quality data and joints to obtain high-quality clean data. The results were then stored in a FASTQ file format. Then, fastqc software (22) was used to perform a quality inspection of the FASTQ file format. BWA software (23) was used to compare the sequencing results of each sample with the reference genome and generated Sam files. Samtools (24) was used to convert Sam files to BAM files and sort them. The featurecounts tool (25) was used to convert BAM files to counts files, which were then used for the construction of expression profiles.

### Differentially Expressed Genes Analysis and Functional Enrichment Analysis

The sequencing data and TCGA data were standardized using rlogTransformation and varianceStabilizingTransformation of the DESeq2 package (26), respectively. DESeq of the DESeq2 package was used for differentially expressed genes’ analysis. Genes with P value adjusted by the false discovery rate (FDR) <0.01 were considered significant, and differentially expressed genes (DEGs) with opposite expressions in sequencing data and TCGA data were defined to be the genes affected by RSV in the treatment of breast cancer. In terms of genes affecting RSV in the treatment of breast cancer, an enrichment analysis was performed using enrichGO and enrichKEGG of the clusterProfiler package (27). The pathway with a P Value <0.05 was considered to be significant.

### Gene Set Variation Analysis (GSVA) and Correlation Analysis

In regard to the enrichment results of the KEGG pathway, the expression spectra of the KEGG pathway were made using gsva of the GSVA package (28), which were used to visualize heat maps. In addition, ggcor of the ggcor package was used to visualize the correlation pathway map.

### Predicting the Docking Model of Resveratrol and POLD1 and the Construction of a Comprehensive Regulation Network

The PDB files for the five POLD1 domains (6s1m, 6s1n, 6s1o, 6tny, 6tnz) (29) were downloaded from the Protein data bank database (PDB, http://www.rcsb.org/pdb/results/results.do?tabtoshow=Current&qrid=9D5B948E) (30). The sdf file of RSV was downloaded from the PubChem database (https://pubchem.ncbi.nlm.nih.gov/) and converted to pdb using Open Babel (31). Then, AutoDockTools (32) was used to predict the docking model of resveratrol and POLD1, eventually attaining the pdbqt file. Open Babel was used to convert pdbqt files into pdb files and visualize the molecular docking results using PyMOL (33). In addition, based on the String database (34), a comprehensive regulation network of the POLD1-targets-pathway was constructed, which was visualized using Cytoscape (35).

### Effect of RSV on POLD1 Expression Gradient

The MDA-MB-231 cells in logarithmic growth phase were adjusted to a concentration of 5 × 10^4^ cells/well into 6-well plates and were inoculated in 6-well plates containing 10% FBS serum DMEM. Subsequently, the MDA-MB-231 cells were placed in a 37 °C, 5% CO_2_ incubator and incubated until 80% confluence, and the blank DMEM medium was used to starve overnight. RSV was divided according to different treatment concentrations into 0, DMSO, 12.5, 25, 50, 100, and 200µM groups, and the MDA-MB-231 cells were treated for 24 h. The cells of each group were collected in a 1.5 ml EP tube, lysed with RIPA cell lysate, and the protein concentration was measured by BCA. The experimental protein samples were then adjusted to the same concentration with RIPA. The protein samples were loaded for 1.5 h following electrophoresis and transferred to a membrane blocked with a 5% skim milk powder blocking solution for 2 h. The primary antibody was incubated overnight (4 °C), and on the next day, it was dipped in TBST for 1 h, which was then incubated with the corresponding secondary antibody for 2 h. Subsequently, it was immersed in TBST for 45 min, and developed with an ECL chemiluminescence developer (Beyotime Biotechnology, China).

### POLD1 Overexpression Transfects Verification

MDA-MB-231 cells in logarithmic growth phase were inoculated into 6-well plates at 5 × 104 cells/well. After 12 h of culturing, POLD1 over-expressing recombinant lentivirus (pCMV-EGFP-POLD1) was added. In addition, a control group and no-load negative control group without viruses were established, and 2 ug/L virus enhancement solution was added at the same time. After 12 h, the medium was changed to a fresh complete medium, and following 72 h, puromycin (2 mg/L) was added for screening. After 1 week, the concentration of puromycin was observed to be reduced by half, and the screening was continued for 1 week. MDA-MB-231/POLD1- Overexpression (OE) and MDA-MB-231/POLD1- Normal control (NC) cell lines were obtained for the subsequent experiments. Transfection efficiency was determined by fluorescence, WB and qPT-PCR.

### Detection of Apoptotic Protein in POLD1 Overexpressing MDA-MB-231 Cells Under RSV Treatment

This experiment was divided into the control group, RSV group and RSV + POLD1-OE group. The protein was extracted 24 h following RSV treatment of the MDA-MB-231 cells. The protein expression levels of POLD1, PARP1, Cleaved-PARP1, PCNA, BCLl-2, and Cleaved-caspase 3 were detected *via* WB. The POLD1 antibody was acquired from Abcam (USA), and GAPDH, Bcl-2, PCNA, PARP1, and cleared Caspase3 antibody were obtained from CST (United States).

### Detection of Apoptotic in POLD1 Overexpressing MDA-MB-231 Cells Under RSV Treatment

The cells in logarithmic growth phase were taken and adjusted to a cell concentration of 5 × 10^4^/ml, after which they were inoculated in 96-well plates (200 µl per well). Next, they were divided into 5 groups with 5 replicates in each group and incubated overnight in a 37 °C, 5% CO_2_ incubator. After the cells adhered, the medium was removed, and the experiment was divided into a control group, a virus no-load control group NC, a no-load control group NC + RSV group, a POLD1 overexpression + RSV group, and a POLD1 overexpression group. Incubation continued under the same conditions for 36 h, after which 10 µl of CCK8 solution was added to each well, and incubation was continued for 3 h. A microplate reader was then used to measure absorbance (A) at 450 nm, and the survival rates of the four groups of cells were compared. The experiment was repeated three times, and the experiment was divided into control group, a virus no-load control group NC, a POLD1-OE group, a POLD1-OE + RSV group, and a no-load control group NC + RSV group. After 36 h of processing, detection was performed using flow cytometry using the AnnexinV APC-7AAD kit (Becton, Dickinson and Company, USA) double standard method.

### Animal Experiments

Nine nude mice were divided into three groups (n = 3) by random grouping into the blank control (Ctrl), RSV, and RSV + POLD1-OE groups. MDA-MB-231 and MDA-MB-231/POLD-OE cells in logarithmic growth-phase were prepared as a suspension of 1 × 107 cells per ml, and 0.1 ml was inoculated subcutaneously into the right lateral axillae of the nude mice. After pinching, the pinholes were clamped with tweezers to prevent cell fluid from leaking. The injection site was observed for ulceration and swelling daily and was ready for use after about 1 week of subcutaneous inoculation. The solvent in the control group was intraperitoneally injected with a 100 µl mixture of PEG400: DMSO: sterile deionized water [1: 1: 3]. The RSV group and the RSV + POLD1-OE group were then injected intraperitoneally with 25 mg/kg RSV containing PEG400: DMSO: sterile deionized water [1: 1: 3] mixed solution (36, 37). After subcutaneous tumorigenesis (generally on the 7th day), the drug was given every other day 8 times.

### Xenograft Assessment, Histopathology, and Immunohistochemistry Assay

During the administration period, the body weight of the nude mice was measured every 4 days, and the longest diameter (a) and shortest diameter (b) of the tumor were measured with a Vernier caliper. Accordingly, 48 h after the last administration, the experimental animals were euthanized, the tumor was weighed, and the tumor inhibition rate was calculated. The tumor tissues were fixed in a 10% formaldehyde solution for 24h and were subsequently fixed in formalin and embedded in paraffin. Sections of the dissected biopsies were stained with hematoxylin and eosin (H&E) using standard procedure and were examined under light microscopy. Paraffin-embedded sections were stained with anti-PCNA (Abcam, USA), anti-Ki-67 (Maixin, China), and anti-Cleaved-Caspase3 monoclonal antibody (CST, USA). HRP-conjugated anti-mouse secondary antibody was added and detected according to the manufacturer’s protocol (Maixin, China). Moreover, the imageJ software was used for image analysis.

### Statistical Analysis

The data was displayed as mean ± standard deviation (x ± s). One-way analysis of variance (One-Way ANOVA) was used for comparison between groups, least significant difference (LSD) test was used for pairwise comparison when variance was uniform, and Dunnett’s T3 test was used for uneven variance. The two groups of data were analyzed by paired T test, and P<0.05 was considered as statistically significant. All data were analyzed using SPSS for Windows 17.0 (Chicago, Illinois, USA).

## Results

### Resveratrol’s Therapeutic Potential in Triple Negative Breast Cancer

**Figure 2:**
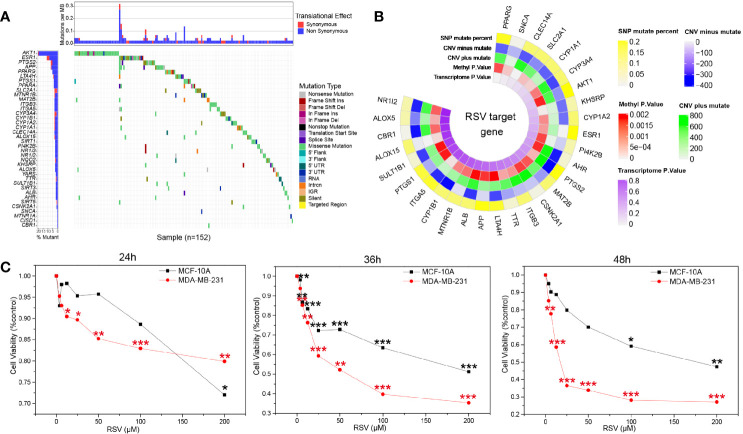
Resveratrol treatment potential in breast cancer. **(A)** Waterfall map of resveratrol target in drugbank and resveratrol target domain protein in protein data bank (TCGA download breast cancer data). **(B)** Multiomics circplot of resveratrol target in drugbank and resveratrol target domain protein in protein data bank (TCGA download breast cancer data). **(C)** Resveratrol inhibited the survival of MDA-MB-231 and MCF-10A cells at different concentrations (24 h, 36 h, 48 h). *p<0.05, **p<0.01, ***p<0.001.

Assessment of the RSV target demonstrated that the target mutation rate of RSV was low (**Figure 2A**), suggesting fewer mutations at the site of action of RSV are present. Circplot of RSV multiomics suggested that the target proteins of RSV may serve as potential drivers for TNBC (**Figure 2B**). CCK8 showed (**Table S1**) that different concentrations of RSV could reduce the survival rate of MDA-MB-231 and MCF-10A. Moreover, with the increase in RSV concentration, the cell viability decreased gradually with increased concentrations of RSV. By comparing cells treated with different concentrations of RSV at 24 h, 36 h, and 48 h, a significant difference was found between the relative survival rate of TNBC cells (**Figure 2C**). When the RSV concentration was 25µM, 50µM, and 100µM, the survival rate of MDA-MB-231 cells and MCF-10A cells had the largest difference. However, at 25 µM concentration (36h), MDA-MB-231 cells had a higher survival rate and slower killing effect. At 100 µM concentration (36 h), the survival rate of MCF-10A cells was lower. The relative survival rate of MDA-MB-231 cells was 52.24% ± 7.03% after 36 h of treatment with 50 µM, suggesting 50 µM was the optimal treating concentration of RSV.

### Effects of Resveratrol on MDA-MB-231 Cells

**Figure 3:**
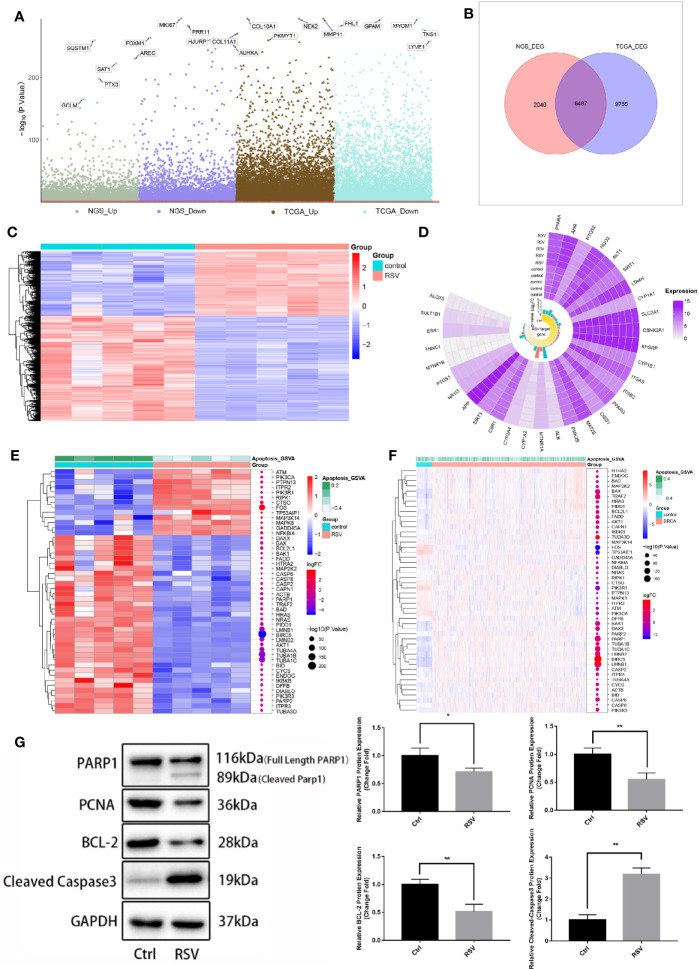
Genes affecting resveratrol in the treatment of MDA-MB-231 cells. **(A)** Manhattan map. The differential expression analysis of the sequencing data of 50 μM resveratrol-treated MDA-MB-231 cell samples -control samples and the differential expression analysis of breast cancer samples - control samples of the TCGA database are respectively displayed. **(B)** Venn diagram. The intersection in the figure is the gene with the opposite differential expression, which is defined as the gene affected by resveratrol in the treatment of breast cancer. **(C)** Expression heat map of genes affected by resveratrol in breast cancer treatment. **(D)** Circplot of resveratrol target in drugbank and resveratrol target domain protein in protein data bank (RNA sequencing data). **(E)** Apoptotic pathway-differential gene expression heat map (RNA sequencing data). **(F)** Apoptotic pathway-differential gene expression heat map (TCGA download breast cancer data). **(G)** Expression of apoptosis-related proteins in MDA-MB-231 cells after optimal resveratrol treatment (PARP1: *P=0.03, PCNA: **P=0.008, BCL-2: **P=0.006, Cleaved Caspased 3: **P=0.001).

To further identify genes affected by RSV in the treatment of breast cancer, the RNA of MDA-MB-231 cells treated with an optimal concentration for RNA sequencing were extracted. The results of the differentially expressed genes analysis (**Figure 3A**) showed that compared to MDA-MB-231 cells without RSV treatment, 8,527 DEGs in MDA-MB-231 cells treated with RSV were present. Additionally, compared to control samples, the BRCA samples had 16,241 DEGs. After comparison, 6,487 DEGs were identified as TNBC genes affected by RSV (**Figure 3B**). **Figure 3C** showed that RSV-treated MDA-MB-231 cells possessed distinct hierarchical clustering from control MDA-MB-231 cells (**Figure 3C**). In addition, the Circplot of RSV targets showed that the expression of some RSV targets changed little after treatment (**Figure 3D**). The heat map of gene expression in the apoptotic pathway illustrates that apoptotic genes were generally elevated in TNBC cells treated with RSV (**Figures 3E, F**). Furthermore, apoptosis-related proteins (PARP1, PCNA, and BCL-2) were detected, and POLD1 and PARP1 expression of MDA-MB-231 cells treated with the optimal concentration of RSV verified the mechanism of TNBC cell apoptosis. Western Blot (**Figure 3G**, **Table S2**) demonstrated that after 24 h of RSV (50uM) treatment, the protein expressions of PARP1 (P<0.05), PCNA (P<0.05), and BCL-2 (P<0.05) were significantly lower than those of the control group, and levels of both Cleaved-PARP1 and Cleaved-Caspase3 (P<0.05) were found to be increased.

### Biological Processes and Pathways of Resveratrol in Treating TNBC

**Figure 4:**
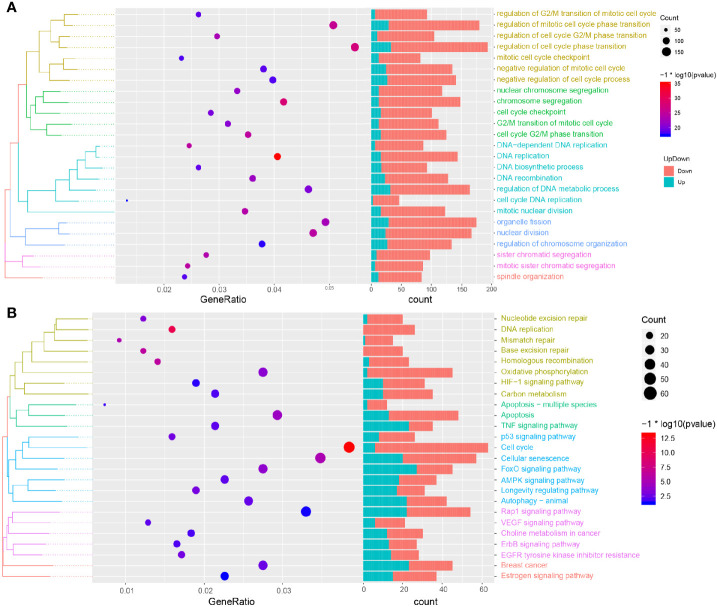
Functional enrichment analysis and GSEA analysis. **(A)** Biological processes involved in resveratrol affected genes. **(B)** KEGG pathways involved in resveratrol affected genes.

In order to further explore the biological process and KEGG pathway affected by RSV in the treatment of TNBC, a functional enrichment analysis was conducted for genes affected by RSV in the treatment of TNBC. The results showed (**Figure 4A**) that genes affected by RSV in TNBC cells were mainly involved in biological processes such as the regulation of mitotic cell cycle phase transition, regulation of cell cycle phase transition, chromosome segregation, DNA replication, and cell cycle DNA replication. Moreover, these genes were mainly involved in the KEGG pathways such as autophagy − animal, MAPK signaling pathway, apoptosis, p53 signaling pathway, and the cell cycle (**Figure 4B**). Most genes affected by resveratrol were found to be enriched in apoptosis related pathways, suggesting that resveratrol can promote the apoptosis of TNBC cells.

### POLD1 Acts as an Intermediary Mediator of MDA-MB-231 Cell Apoptosis

**Figure 5:**
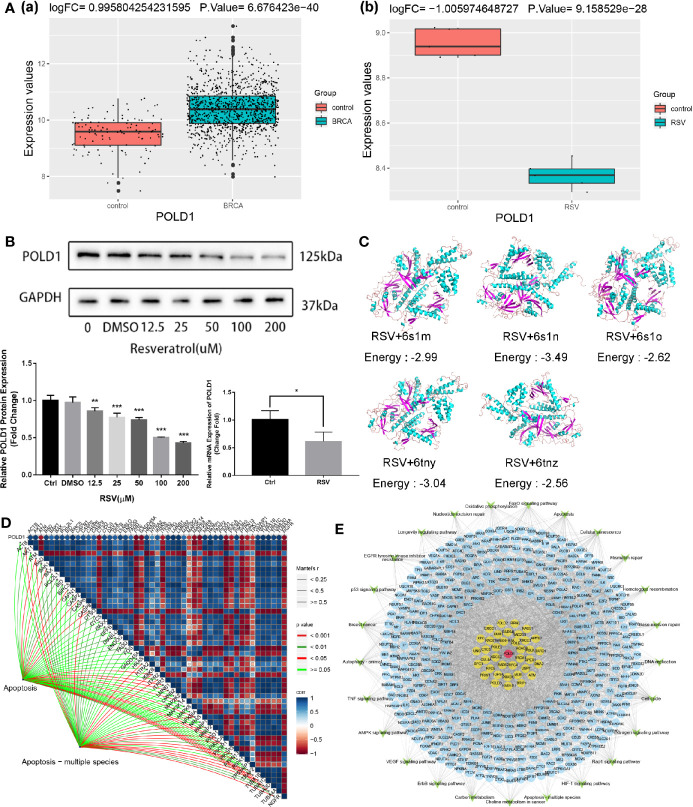
Comprehensive regulatory network for resveratrol in treating MDA-MB-231 cells. **(A)** (a) POLD1 expression in BRCA samples of TCGA database. (b) POLD1 expression in MDA-MB-231 cells samples after resveratrol treatment. **(B)** Protein expression of POLD1 after treatment with different concentrations of resveratrol (*P=0.043, **P=0.004, ***P<0.001). **(C)** Combined prediction model of resveratrol and POLD1 domain. **(D)** POLD1-differential apoptotic pathway gene-apoptotic pathway correlation pathway map. **(E)** Comprehensive regulatory network diagram. Red represents POLD1, yellow represents genes that interact with POLD1, blue represents genes that are affected by other resveratrol treatments for TNBC, and green represents pathways.

Previous studies have suggested that POLD1 may be associated with the malignant survival of tumor cells and that POLD1 is generally highly expressed in BRCA tissues (38). In this study, this result was verified in BRCA data of TCGA database [**Figure 5A(a)**]. Moreover, we also found that POLD1 was downregulated in MDA-MB-231 cells treated with RSV [**Figure 5A(b)**], suggesting it may serve as an intermediary for RSV in the treatment of TNBC. As shown in **Figure 5B** (**Table S3**), after RSV treatment, protein and mRNA expression of POLD1 gradually decreased with the increase in treatment concentration. The molecular docking results of RSV and POLD1 indicated (**Figure 5C**) that the docking energy with five domains of POLD1 (6s1m, 6s1n, 6s1o, 6tny and 6tnz) was generally less than 0 kcal/mol, suggesting that POLD1 may serve as a potential target of RSV. In addition, the results of the correlation pathway map (**Figure 5D**) showed that POLD1 was related to most genes in the apoptotic pathway, suggesting that RSV may inhibit TNBC by reducing the expression of POLD1 and mediating TNBC’s potential mechanisms of apoptosis. Finally, a comprehensive regulatory map of TNBC cell genes treated by RSV was constructed (**Figure 5E**). The network diagram illustrates that TNBC cells treated by RSV mainly affect the protein encoded by other genes through POLD1, which then affect different pathways.

### Overexpression of POLD1 Reduces Apoptosis of MDA-MB-231 Cell Induced by Resveratrol

**Figure 6:**
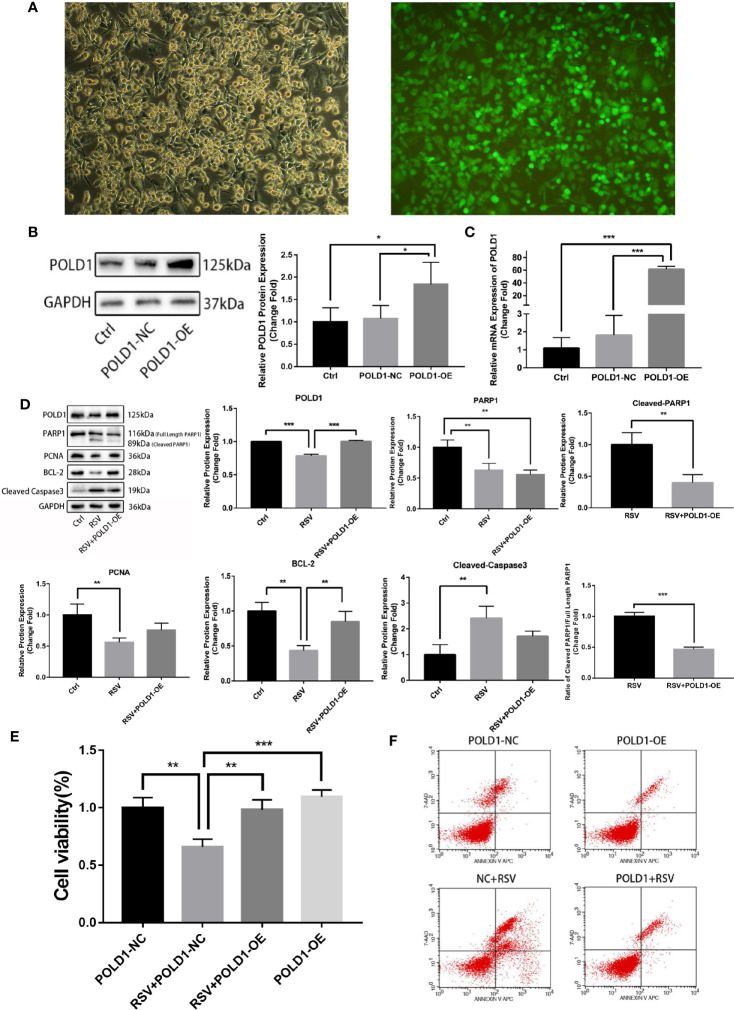
POLD1 rescue experiment **(A)** Immunofluorescence of POLD1 transfected with lentivirus. **(B)** POLD1 protein expression after transfection (control and POLD1-NC: *P=0.035, POLD1-NC and POLD1-OE: *P=0.048). **(C)** Gene expression of POLD1 after transfection (control and POLD1-NC: **P=0.007, POLD1-NC and POLD1-OE: **P=0.007). **(D)** Apoptosis indicators of breast cancer cells after transfection (POLD1:***P<0.001, PARP1: control and RSV+POLD1-OE: **P=0.004, RSV and RSV+POLD1-OE: **P=0.001, Cleaved PARP1/Full Length PARP1: ***P<0.001, PCNA: **P=0.004, BCL-2:control and RSV: **P=0.001, RSV and RSV+POLD1-OE: **P=0.006, Cleaved Caspased 3: **P=0.003),. **(E)** CCK8 detection of resveratrol treated breast cancer cell survival after transfection (**P=0.001, ***P<0.001). **(F)** Flow cytometry detection of resveratrol-treated apoptosis of breast cancer cells after transfection (on the two-parameter scatter diagram of flow cytometry, the lower left quadrant represents living cells; the upper left quadrant represents necrotic cells; the lower right quadrant represents apoptotic cells (late apoptotic cells), and the upper right quadrant represents early apoptotic cells).

To further verify whether POLD1 is an intermediary for RSV in the treatment of TNBC, a rescue experiment of POLD1 overexpression (OE) was performed. The results of the POLD1 lentivirus-transfected cells showed that the transfection efficiency was over 90% (×100-fold) (**Figure 6A**). WB and qRT-PCR showed that the expression of POLD1 protein (nearly 2 times) and mRNA (about 25 times) in POLD1-OE group were highly expressed (**Figures 6B, C**, **Table S4**). In addition, the proteins after RSV treated MDA-MB-231 cells for 24 h were extracted, and protein was detected in expression levels of POLD1, PARP1, Cleaved-PARP1, PCNA, BCL-2, Cleaved-Caspase3, and other proteins using WB. Accordingly, the results demonstrated that the protein expression level of POLD1 in the RSV + POLD1-OE group was significantly higher than that in the RSV group (P<0.01), and the expression level of BCL-2 (P<0.05) was also significantly higher than that in the RSV group (**Figure 6D**, **Table S5**). However, the expression of Cleaved-PARP1 (P<0.01) and Cleaved-Caspase3 (P<0.05) was significantly lower. Although the expression of full length PARP1 decreased, the ratio of Cleaved-PARP1/full length PARP1 (P<0.01) decreased significantly. Overexpression of POLD1 and RSV treatment can synergistically lead to a further reduction in PARP1 expression, however, POLD1 overexpression can reduce the ratio of Cleaved-PARP1/full length PARP1, indicating apoptosis. CCK8 showed (**Figure 6E**, **Table S6**) that RSV could significantly promote MDA-MB-231 cell apoptosis, while POLD1 overexpression could slightly increase the proliferation ability of MDA-MB-231 cells. Moreover, overexpression of POLD1 could significantly reduce RSV’s pro-apoptotic effect on MDA-MB-231 cells. At the same time, flow cytometry demonstrated (**Figure 6F**) that RSV could significantly promote MB-231 cell apoptosis, while overexpression of POLD1 could reduce the apoptotic effect of RSV on MB-231 cells.

### Resveratrol Mediates MDA-MB-231 Cell Apoptosis Through a POLD1-Driven Apoptosis Pathway

**Figure 7:**
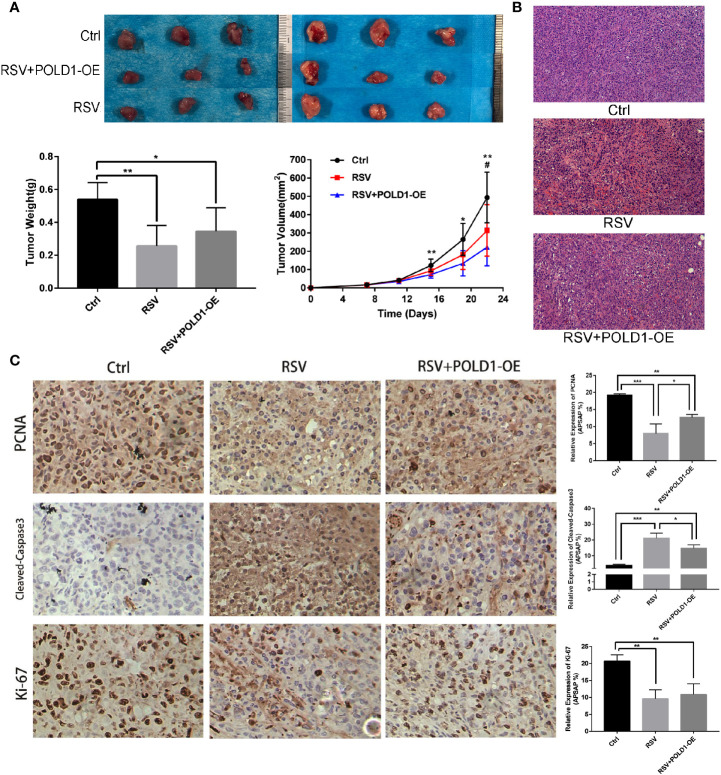
Identification of RSV induced apoptosis in MDA-MB-231 cells *in vitro* and *in vivo*. **(A)** Breast cancer proliferation after resveratrol and resveratrol + POLD1-OE treatment in mouse model. **(B)** Apoptosis was detected by hematoxylin-eosin (HE) staining after Resveratrol and Resveratrol + POLD1-OE treatment in mice. **(C)** Immunohistochemistry of related apoptotic proteins after resveratrol and resveratrol + POLD1-OE treatment in mice. *p<0.05, **p<0.01, ***p<0.001.

Animal experiments showed that RSV has a direct inhibitory effect on TNBC in nude mouse models, while overexpression of the POLD1 gene was found to reduce the inhibitory effect of RSV on tumors. Compared to the control group (**Figure 7A**, **Table S7**), the RSV and RSV + POLD1-OE groups had a smaller final tumor volume (493.66 ± 138.30mm3 vs. 222.70 ± 102.55mm3 and 313.48 ± 140.93mm3, P <0.01 P >0.05) and lower wet weight (0.54 ± 0.10 g vs. 0.26 ± 0.13 g and 0.34 ± 0.14 g, P <0.01, P <0.05). HE staining showed that the tumor tissue of the RSV group had obvious necrosis, while the RSV + POLD1-OE group had less fibrous tissue than the RSV group (**Figure 7B**). In addition, immunohistochemistry illustrated (**Figure 7C**, **Table S8**) that RSV can reduce the expression of PCNA (P<0.001) and Ki-67 (P<0.01) and increase the expression of Cleaved-Caspase3 (P<0.001). However, the POLD1 overexpression group antagonized these inhibitory or promotive effects (× 200-fold), signifying that POLD1 was an intermediary for RSV in treating MDA-MB-231 cells. Mechanistically, RSV may drive the apoptosis gene by reducing the expression of POLD1, activating the apoptosis pathway in the apoptosis of TNBC cells (**Figure S1**).

## Discussion

RSV has previously been found to inhibit breast cancer cells by hindering the proliferation of breast cancer cells through its cell cycle and inducing apoptosis (39). Moreover, RSV possesses abrogate stemness properties and can reduce the expression of self-renewal signaling molecules in stem-like breast cancer cells (40). However, its anti-cancer mechanism on TNBC has not been clarified. In this study, *via* CCK8 experiments, the survival rates of MDA-MB-231 and MCF-10A were observed to gradually decrease following treatment with different concentrations of RSV, indicating that RSV can inhibit the survival of TNBC cells. By comparing the survival rates of MDA-MB-231 and MCF-10A treated by different concentrations of RSV, 50 μM was identified to be the optimal concentration of RSV, and MDA-MB-231 cells treated with this concentration were extracted for RNA sequencing.

According to the results of the differentially expressed gene analysis, the results of the two DEG groups were compared, and DEGs were defined with opposite expressions in the two groups as genes affected by RSV treatment in TNBC cells. Moreover, WB showed that apoptosis related proteins like Cleaved-Caspase 3 and BCL-2 were detected in MDA-MB-231 treated by RSV. In tumor therapy, apoptosis is a well-known mechanism of cell death, involving the activation of caspases (41). Caspases are key mediators in programmed cell death or apoptosis, where caspase 3 catalyzes the specific cleavage of many key cellular proteins (42). Additionally, BCL2 is originally characterized with respect to their roles in controlling outer mitochondrial membrane integrity and apoptosis (43, 44). The high expression of BCL2 is closely related to tumorigenesis, and the overexpression of BCL2 family proteins promotes the occurrence and development of tumors, while its inhibition is related to anti-tumor characteristics (45, 46). These results indicate that RSV can reduce the survival rate of TNBC cells by promoting apoptosis. To this effect, a functional enrichment analysis showed that genes affected by the corresponding RSV in the treatment of TNBC were significantly involved in both the apoptotic signal pathway and P53 signal pathway. P53 is a tumor suppressor protein that regulates the expression of various genes including apoptosis, growth inhibition, inhibition of cell cycle progression, differentiation, and accelerated DNA repair (47, 48). In general, p53 can regulate cell growth by promoting apoptosis and DNA repair under stress. However, when p53 mutates, it loses its regulatory function, leading to abnormal cell proliferation and tumor progression (49). Apoptosis refers to programmed cell death, resulting in the orderly and effective removal of damaged cells, such as DNA damage or cell damage caused during development (50, 51). Apoptosis may be triggered *via* intracellular signals (such as genotoxic stress) or external signals (such as the binding of ligands to cell surface death receptors) (52, 53). Deregulation of apoptotic cell death mechanisms is a feature of cancer. Here, the genes of TNBC cells treated by RSV were found to be significantly involved in signal pathways like apoptosis and P53, suggesting that RSV can activate apoptosis of TNBC cells.

Previous studies have shown that POLD1 may be associated with the malignant proliferation of tumor cells and that a high expression of POLD1 is associated with the poor prognosis of breast cancer (38). The present study found that the low expression of POLD1 in TNBC cells treated by RSV may be related to the treatment of RSV. Further experimentation demonstrated that as the RSV treatment concentration rose, the protein expression of POLD1 in TNBC cells gradually decreased. Molecular docking suggested that RSV may bind to POLD1, and POLD1 possesses a strong correlation with DEGs in the apoptotic pathway. Hence, a comprehensive regulatory landscape of RSV-treated TNBC cells was constructed. According to the network, POLD1 was observed to interact with ATM, which is a DNA damage response gene commonly mutated in cancer. ATM activation serves as a key factor in balancing between aging and apoptosis as well as autophagy (54). In this study, ATM was found to be highly expressed in RSV affected MDA-MB-231 cells, which may be related to its apoptosis promoting mechanism. A rescue experiment was then performed with POLD1 overexpression, demonstrating that the expression levels of POLD1, PCNA, and BCL-2 in MDA-MB-231 cells treated with RSV after the overexpression of POLD1 were higher than those in the RSV treated group, while the levels of Cleaved-Caspase3 and Cleaved-PARP1 reduced. Among them, PCNA was considered to be a molecular marker of proliferation due to its role in replication (55). Here, the expression of PCNA treated with RSV decreased, while the proliferation ability of POLD1 overexpression treatment was relatively high, indicating that POLD1 has an effect on the proliferation of TNBC cells. In addition, PARP1 was discovered to be a molecular target of anti-tumor drugs. In melanoma, breast cancer, lung cancer, and other neoplastic diseases, the expression of PARP1 is often increased (56). Therefore, the corresponding results demonstrate that POLD1 overexpression could reduce the apoptosis effect of RSV on MDA-MB-231 cells. Moreover, CCK8 and flow cytometry revealed that RSV can promote TNBC cell apoptosis, while the overexpression of POLD1 can reduce the apoptosis effect of RSV on MDA-MB-231 cells, further suggesting that RSV can mediate TNBC cell apoptosis by reducing the expression of POLD1.

Further animal experiments showed that compared to the control group, the final tumor volume and wet weight of the RSV and RSV + POLD1-OE groups were smaller. HE staining illustrated that the distribution of cancer cells was sparse, and the proportion of fiber components was observed to be significantly higher. Immunohistochemistry of the animal models showed that RSV could reduce the expression of PCNA and Ki-67 while increasing the expression of Cleaved-Caspase3. However, the POLD1 overexpression group antagonized these inhibitory or promoting effects. Ki-67 is a human nuclear antigen and forms an integral part of cell division in both normal and malignant tissue (57). High expression of Ki-67 serves as a marker for poor prognosis in breast cancer (58, 59), and the obtained results demonstrated that RSV can inhibit the growth of TNBC cells and promote necrosis in cancer cells *in vivo* but was limited in the overexpression of POLD1. This suggested that the overexpression of POLD1 can antagonize the inhibitory effect of RSV on TNBC *in vivo* and serves as an important regulatory target of RSV in the treatment of TNBC.

Although this study explored and identified a potential mechanism for the anticancer effect of RSV on TNBC cells, various limitations exist. First, relatively few samples were used in this experiment, and the results should be verified in larger sample studies. Second, this study used only one triple negative cell line and verified the proliferation and apoptosis of MDA-MB-231 cells treated by RSV at the gene level, protein level and individual animal level. However, the relationship between RSV and POLD1 requires further elucidation. More importantly, although the anti-TNBC effect of RSV has been clarified, due to poor solubility and bioavailability of RSV, as well as adverse reactions, its clinical use has been restricted. Therefore, making RSV into a drug to better treat breast cancer remains to be studied.

## Conclusion

In conclusion, this study determined a potential anticancer mechanism of RSV against TNBC by conducting bioinformatics and experimental studies. Accordingly, RSV was found to promote the apoptosis of TNBC cells by reducing POLD1 expression, thereby activating the respective apoptosis pathways. However, since this study is a basic mechanism study, this method has not yet been applied to human patients.

## Data Availability Statement

We have uploaded the raw data to the Sequence Read Archive database. You can access it through the following link: https://www.ncbi.nlm.nih.gov/sra/PRJNA680177.

## Ethics Statement

The animal study was reviewed and approved by Guangxi Medical University.

## Author Contributions

C-YW and H-ML developed the research design, evaluated all of the results, and were responsible for the article. Z-JL and YW developed the experimental design and analyzed the data. D-DZ, M-XW, H-MJ, D-LH, L-FL, M-JC, and W-PY performed research and contributed to the writing of this paper. All authors contributed to the article and approved the submitted version.

## Funding

The authors disclosed receipt of the following financial support for the research, authorship, and/or publication of this article: This work was financially supported by the National Natural Science Foundation of China (81760346, 81860341), Natural Science Foundation of Guangxi Province (2018GXNSFAA281148, 2020JJA140036), Innovation Project of Guangxi Graduate Education (YCBZ2018041), the Scientific Research & Technology Development Program of Nanning City (20173021-2, 20183037-1, 20191034), the Nanning Excellent Young Scientist Program and Guangxi Beibu Gulf Economic Zone Major Talent Program (RC20180201, RC20190206), Self-financing research Project of Guangxi Health and Family Planning Commission (Z20180679, Z20190139), and the Youth Science Foundation of Guangxi Medical University (GXMUYSF201808).

## Conflict of Interest

The authors declare that the research was conducted in the absence of any commercial or financial relationships that could be construed as a potential conflict of interest.

## Supplementary Material

Supplementary Figure 1 Potential mechanisms of resveratrol promoting apoptosis of triple negative breast cancer cells.

Supplementary Table 1 The result of CCK8 detection of cell viability after RSV treatment.

Supplementary Table 2 Protein expression of PCNA, BCL-2, Cleaved-PARP1 and Cleaved-Caspase3 after RSV treated.

Supplementary Table 3 WB and qRT-PCR of POLD1 after RSV treated.

Supplementary Table 4 Protein expression and mRNA expression of POLD overexpression.

Supplementary Table 5 Rescue experiment of POLD1 overexpression.

Supplementary Table 6 The result of CCK8 detection of cell viability after rescue experiment.

Supplementary Table 7 Weight and volume of nude mouse models after RSV treated.

Supplementary Table 8 PCNA, Cleaved-Caspase3 and Ki67 of immunohistochemistry after RSV treated.
