# Supplementary figures and images for: Resveratrol Mediates the Apoptosis of Triple Negative Breast Cancer Cells by Reducing POLD1 Expression
Source: Front Oncol. 2021 Feb 25;11:569295. doi: 10.3389/fonc.2021.569295 (PMC7970754; doi:10.3389/fonc.2021.569295)

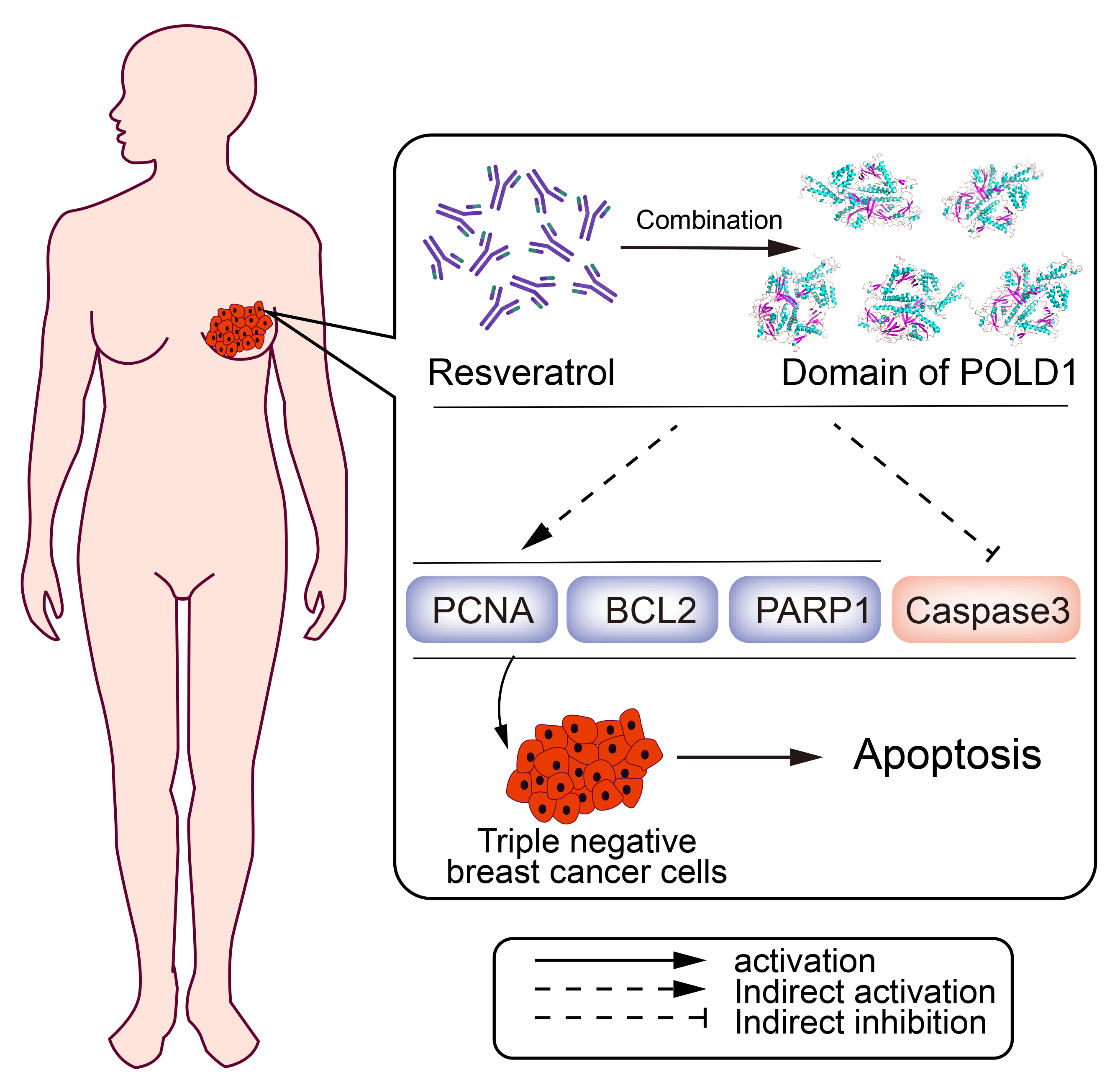

Supplement: Supplementary Figure 1 — Potential mechanisms of resveratrol promoting apoptosis of triple negative breast cancer cells. [file Image_1.jpeg]
